# Eosinophil-to-Platelet Ratio in Comparison With Dyspnea, Eosinopenia, Consolidation, Acidemia, and Atrial Fibrillation (DECAF) Score in Acute Exacerbation of COPD: A Prospective Observational Study

**DOI:** 10.7759/cureus.97807

**Published:** 2025-11-25

**Authors:** Divya R V, Kolla Vinod, Ponnathota Vindhya

**Affiliations:** 1 Department of Pulmonary Medicine, RajaRajeswari Medical College and Hospital, Bengaluru, IND

**Keywords:** acute exacerbation, copd, decaf score, eosinophil-to-platelet ratio, prognostic biomarkers, risk stratification

## Abstract

Aim and background: Acute exacerbations of chronic obstructive pulmonary disease (AECOPD) account for the top three causes of death worldwide. Identifying inexpensive, laboratory-based markers that can readily predict disease severity and short-term outcomes, therefore, remains a clinical priority. This study examined the prognostic utility and association of the eosinophil-to-platelet ratio (EPR) in patients admitted with AECOPD and compared its predictive performance with the Dyspnea, Eosinopenia, Consolidation, Acidemia, and Atrial Fibrillation (DECAF) score.

Methods: A six-month prospective observational study was undertaken at RajaRajeswari Medical College and Hospital, Bengaluru, following institutional ethics approval and written informed consent. Fifty consecutive adults admitted with a clinical diagnosis of AECOPD were enrolled. On admission, a complete blood count was obtained to calculate EPR; receiver operating characteristic (ROC) analysis identified an optimal cutoff value of 0.000019. DECAF scores were obtained. Primary endpoints included in-hospital mortality, intensive care unit (ICU) admission, and type of respiratory failure.

Results: Patients with EPR < 0.000019 had significantly higher in-hospital mortality (25% versus 3.8%), a greater incidence of hypercapnic respiratory failure (70.8% versus 30.7%), and more ICU admissions (58.3% versus 15.3%) compared to those with higher EPR. A strong correlation was observed between low EPR values and high-risk DECAF scores, with 79.1% of low-EPR patients falling into the high-risk category. ROC analysis confirmed the discriminatory ability of EPR for adverse outcomes.

Conclusion: EPR is a statistically and clinically significant predictor of adverse outcomes in AECOPD and shows a strong correlation with DECAF score. It holds promise as a rapid, reliable tool for initial triage.

Clinical significance: EPR, derived from routine hematology, may serve as a quick and affordable bedside marker to assist in early risk stratification and clinical decision-making in patients hospitalized with AECOPD.

## Introduction

Chronic obstructive pulmonary disease (COPD) is a common, relentlessly progressive lung disorder and a major cause of chronic morbidity and mortality throughout the world. The most recent Global Burden of Disease analysis lists COPD as the world's third leading cause of death, responsible for roughly 3.2 million deaths each year [1]. The burden is rising fastest in low- and middle-income countries, where exposure to tobacco smoke and ambient air pollution remains substantial, underscoring COPD's significance as a public health challenge [2]. COPD is a heterogeneous lung condition characterized by chronic respiratory symptoms (dyspnea, cough, sputum production, and/or exacerbation) due to abnormalities of the airways (bronchitis and bronchiolitis) and/or alveoli (emphysema) that cause persistent, often progressive, airflow obstruction [3].

Acute exacerbation of chronic obstructive pulmonary disease (AECOPD) is defined as an event characterized by increased dyspnea and/or cough and sputum that worsens in less than 14 days, which may be accompanied by tachypnea and/or tachycardia and is often associated with increased local and systemic inflammation caused by infection, pollution, or other insults to the airways. Exacerbations accelerate the decline in lung function, increase hospitalization and ventilatory support requirements, and increase short- and long-term mortality risk [3,4]. As such, the frequency and severity of exacerbations have become key prognostic indicators and integral in patient stratification and management strategies [5].

Given COPD's heterogeneity and systemic manifestations, there is a clear need for inexpensive, readily obtainable biomarkers that can predict disease severity, patient outcomes, and therapeutic decisions [6]. Laboratory indices derived from routine blood counts are particularly useful in resource-constrained settings where advanced investigations may not be available. Among these emerging markers, eosinophil- and platelet-based parameters have shown promise for both diagnostic and prognostic purposes [7,8].

The Dyspnea, Eosinopenia, Consolidation, Acidemia, and Atrial Fibrillation (DECAF) score, introduced by Steer et al., offers a pragmatic bedside approach to estimating in-hospital mortality in patients admitted with an acute exacerbation of COPD [9]. The score assigns 1 point each for (i) severe dyspnea assessed with the extended Medical Research Council (eMRC) scale (eMRCD 5a: 1 point, eMRCD 5b: 2 points), (ii) eosinopenia (absolute eosinophil count < 0.05 × 10^9^L⁻¹), (iii) radiographic consolidation, (iv) acidemia (arterial pH < 7.30), and (v) the presence of atrial fibrillation. Summative scores stratify patients into low (0-1), intermediate (2), or high (3-6) mortality risk categories. Owing to its simplicity and robust predictive accuracy across diverse cohorts, the DECAF score has gained widespread clinical adoption [10].

Of particular interest in the DECAF score is the component of eosinopenia, which serves as an indicator of systemic stress and immune suppression. Eosinophils, while traditionally linked to allergic conditions and parasitic infections, play complex roles in host defense and inflammatory regulation [11]. In the context of COPD, their levels in peripheral blood have been shown to fluctuate with disease activity and therapeutic response. Eosinopenia has been associated with severe infections, sepsis, and poor outcomes in critically ill patients [12,13]. During acute exacerbations, low eosinophil counts have been linked with increased bacterial colonization, reduced responsiveness to corticosteroids, and greater severity of illness [14].

Platelets, although classically associated with coagulation, are now understood to be dynamic mediators of inflammation and immune regulation. In COPD, elevated platelet counts have been associated with systemic inflammation, endothelial dysfunction, and oxidative stress [15]. Studies suggest that platelet activation may enhance neutrophilic inflammation, promote microvascular injury, and participate in airway remodeling [16]. Furthermore, thrombocytosis has been associated with increased frequency of exacerbations and higher mortality risk in patients with COPD [17].

The integration of eosinophil and platelet counts into a composite index, the eosinophil-to-platelet ratio (EPR), offers a novel, easily obtainable marker of systemic inflammatory burden. The rationale is that a lower EPR reflects both immunosuppression (eosinopenia) and heightened inflammatory activation (thrombocytosis), serving as a surrogate indicator of disease severity and adverse clinical outcomes [18]. Emerging evidence from sepsis and oncology research has demonstrated the prognostic utility of similar hematologic ratios, such as the neutrophil-to-lymphocyte ratio and platelet-to-lymphocyte ratio [19]. However, data regarding the role of EPR in COPD, particularly during exacerbations, remain sparse.

In the context of acute exacerbations of COPD, where rapid risk stratification is essential for decision-making regarding hospitalization, intensive care unit (ICU) transfer, and initiation of aggressive therapies, EPR could potentially serve as a rapid, inexpensive prognostic marker [20]. Importantly, EPR is derived from tests that are universally available and routinely performed on admission, thus making it a feasible tool even in resource-constrained environments.

This prospective, observational study set out to clarify how the eosinophil-to-platelet ratio (EPR) relates to the DECAF score in adults admitted with acute COPD exacerbations. Our first aim was to see whether EPR, on its own, could identify patients at higher risk of in-hospital death, respiratory failure, or ICU transfer. We also explored whether lower EPR values line up with higher DECAF scores and poorer outcomes, evidence that would position the ratio as an easy-to-obtain adjunct, or even an alternative, to existing prognostic tools.

## Materials and methods

Study design and duration

This study is a prospective observational cohort study conducted on 50 consecutive patients admitted to the wards and ICU of the respiratory medicine department of RajaRajeswari Medical College and Hospital, Bengaluru, from March 2025 to June 2025. The study was conducted to test the eosinophil-to-platelet ratio (EPR) to predict the following clinical outcomes in adults admitted with an acute exacerbation of COPD (AECOPD): transfer to the ICU, type of respiratory failure, and in-hospital death. We also examined how closely EPR aligns with the well-validated DECAF score.

Ethical approval and consent

The Institutional Ethics Committee of RajaRajeswari Medical College and Hospital (RRMCH) reviewed the protocol on February 28, 2025, and issued written approval on March 12, 2025. All procedures adhered to the Declaration of Helsinki and the Indian Council of Medical Research guidelines for human research. Before enrolment, every participant received a thorough explanation of the study and signed an informed consent form. Patient data were coded, anonymized, and handled in strict confidence.

Objective

The primary objective was to correlate the eosinophil-to-platelet ratio with the severity of patients in comparison with the DECAF score. Secondary objectives included assessing mortality in patients with a low eosinophil-to-platelet ratio and evaluating complications such as respiratory failure in comparison with the eosinophil-to-platelet ratio.

Inclusion criteria

The study included patients more than 45 years old who satisfied the criteria of AECOPD according to the Global Initiative for Chronic Obstructive Lung Disease (GOLD) guidelines 2025 [21].

Exclusion criteria

Patients were excluded if they were on corticosteroids before admission, below 45 years of age, or diagnosed with allergy and parasitic infection, or eosinophilic disease.

Data collection and measurements

On admission, each participant underwent a thorough bedside evaluation: we took a detailed history, recorded vital signs, performed physical examination, and documented presenting symptoms. Baseline investigations comprised a complete blood count, arterial blood gas analysis, chest radiography, and a 12-lead electrocardiogram (ECG). The eosinophil-to-platelet ratio (EPR) was then derived by dividing the absolute eosinophil count by the platelet count, both obtained from an automated hematology analyzer (Sysmex XN-1000; Sysmex, Kobe, Japan).

The DECAF score was calculated for each patient using five variables: dyspnea severity based on the extended Medical Research Council (eMRCD) dyspnea scale, with a score of 5a awarded 1 point and 5b awarded 2 points; eosinopenia (absolute eosinophil count < 0.05 × 10^9^/L) awarded 1 point, presence of radiological consolidation awarded 1 point, arterial acidemia (pH < 7.3) awarded 1 point, and atrial fibrillation, either from ECG or medical history, awarded 1 point [8]. The score was then stratified into low-risk (0-1), intermediate-risk (2), and high-risk (3-6) categories as per previously validated studies [9].

Statistical analysis

Data were transcribed into Microsoft Excel (Microsoft Corp., Redmond, WA) and analyzed using IBM SPSS Statistics version 26.0 (IBM Corp., Armonk, NY). Continuous variables are presented as mean ± standard deviation (SD) or median with interquartile range (IQR), depending on the normality of distribution, while categorical variables are summarized as counts and percentages. The prognostic capacity of EPR for in-hospital mortality was examined using receiver operating characteristic (ROC) curve analysis. In-hospital mortality was used as the primary endpoint for ROC analysis. The optimal cutoff value of 0.000019 was identified using the Youden index (sensitivity + specificity - 1), which maximizes the sum of sensitivity and specificity. The ROC curve yielded an area under the curve (AUC) of 0.78 (95% confidence interval (CI): 0.64-0.92, p = 0.002), demonstrating good discriminatory ability. At this threshold, sensitivity was 85.7% and specificity was 75% for predicting in-hospital mortality. This cutoff divided participants into high-risk (EPR < 0.000019) and low-risk (EPR ≥ 0.000019) groups. Internal validation was not performed due to the limited sample size; external validation in larger cohorts is needed before clinical implementation.

Equipment and software used

The hematologic parameters were obtained using the Sysmex XN-1000 automated analyzer. Arterial blood gas analyses were performed using the Roche Cobas b 123 POC system (Roche Diagnostics, Germany). Chest X-rays were acquired using the Philips DigitalDiagnost C90 system (Philips Healthcare, Netherlands). All statistical analyses were carried out using IBM SPSS Statistics for Windows version 26.0.

## Results

A total of 50 patients were included in the study after satisfying the inclusion criteria. The majority were male patients (72%), and the mean age was 64.3 ± 9.1 years. The baseline characteristics, including demographic data, smoking history, symptom duration, comorbidities, and initial vital parameters, are summarized in Table 1.

**Table 1:** Baseline characteristics of the study participants (N = 50) Data presented as mean ± standard deviation for continuous variables and percentages for categorical variables. Comorbidities were identified from medical history at admission. COPD: chronic obstructive pulmonary disease, SpO_2_: peripheral oxygen saturation

Parameter	Value
Total patients (number)	50
Mean age (years)	64.3 ± 9.1
Male (%)	72%
Female (%)	28%
Smokers (%)	80%
Mean duration of COPD (years)	6.2 ± 2.5
Comorbidities (hypertension) (%)	46%
Comorbidities (diabetes mellitus) (%)	34%
Comorbidities (coronary artery disease) (%)	22%
Average SpO_2_ at admission (%)	88.6 ± 3.9
Mean respiratory rate (/minute)	24.7 ± 2.3
Mean heart rate (/minute)	98.5 ± 6.4

The receiver operating characteristic (ROC) curve analysis identified an optimal eosinophil-to-platelet ratio (EPR) cutoff value of 0.000019 for predicting in-hospital mortality. Patients were stratified into two groups accordingly: Group A (EPR ≥ 0.000019) and Group B (EPR < 0.000019). Among the study population, 26 (52%) patients fell into Group A, while 24 (48%) patients were in Group B. The clinical characteristics and outcomes across the two groups are shown in Table 2.

**Table 2:** Comparison of clinical outcomes between high EPR (≥0.000019) and low EPR (<0.000019) groups Data presented as percentages with absolute numbers in parentheses. Statistical comparisons performed using the χ² test or Fisher's exact test. Hypercapnic respiratory failure defined as PaCO2 > 45 mmHg with respiratory acidosis. EPR: eosinophil-to-platelet ratio, PaCO2: partial pressure of arterial carbon dioxide, ICU: intensive care unit, DECAF: Dyspnea, Eosinopenia, Consolidation, Acidemia, and Atrial Fibrillation

Clinical parameter	EPR ≥ 0.000019 (n = 26)	EPR < 0.000019 (n = 24)	p-value
In-hospital mortality (%)	3.8% (1/26)	25% (6/24)	<0.05
Hypercapnic respiratory failure (%)	30.7% (8/26)	70.8% (17/24)	<0.01
ICU admission (%)	15.3% (4/26)	58.3% (14/24)	<0.01
DECAF score (high risk 3-6) (%)	7.6% (2/26)	79.1% (19/24)	<0.001
Need for ventilatory support (%)	26.9% (7/26)	66.6% (16/24)	<0.01

When comparing outcomes, patients with EPR < 0.000019 (Group B) exhibited significantly worse clinical profiles. The mortality rate was markedly higher in Group B, with six out of 24 (25%) patients succumbing during hospitalization, compared to only one out of 26 (3.8%) patients in Group A. The difference in mortality was statistically significant (p < 0.05). Additionally, hypercapnic respiratory failure was more prevalent in Group B, with 17 out of 24 (70.8%) patients developing elevated PaCO_2_ levels requiring non-invasive or invasive ventilatory support. In contrast, only eight out of 26 (30.7%) patients in Group A developed similar complications, as shown in Table 3.

**Table 3:** Distribution of DECAF score risk categories stratified by EPR Data presented as absolute numbers with percentages in parentheses. DECAF categories: low risk (0-1), intermediate risk (2), and high risk (3-6). Statistical analysis showed a significant association between low EPR and high-risk DECAF categories (p < 0.001). DECAF: Dyspnea, Eosinopenia, Consolidation, Acidemia, and Atrial Fibrillation, EPR: eosinophil-to-platelet ratio

DECAF score category	EPR ≥ 0.000019 (n = 26)	EPR < 0.000019 (n = 24)
Low risk (score 0-1)	20 (76.9%)	2 (8.3%)
Intermediate risk (score 2)	4 (15.3%)	3 (12.5%)
High risk (score 3-6)	2 (7.6%)	19 (79.1%)

The association between EPR and DECAF score classification was also analyzed. Patients with low EPR values were found to have disproportionately high DECAF scores. Specifically, 19 out of 24 (79.1%) patients in Group B had a DECAF score in the high-risk range (score 3-6), while only two (7.6%) patients in Group A were in the same category. Conversely, 20 out of 26 (76.9%) patients in Group A were classified as low-risk (score 0-1), and only two (7.6%) patients fell into the high-risk group. This trend demonstrates a clear inverse relationship between EPR and DECAF score category, as given in Table 4.

**Table 4:** Clinical outcomes according to DECAF score risk categories Data presented as absolute numbers with percentages in parentheses. Respiratory failure includes both hypoxemic and hypercapnic types requiring ventilatory support. Progressive increase in adverse outcomes validates the DECAF score utility. DECAF: Dyspnea, Eosinopenia, Consolidation, Acidemia, and Atrial Fibrillation, ICU: intensive care unit

DECAF score category	ICU admission (%)	Respiratory failure (%)	In-hospital mortality (%)
Low risk (score 0-1)	2 (7.4%)	3 (11.1%)	0 (0%)
Intermediate risk (score 2)	3 (33.3%)	4 (44.4%)	1 (11.1%)
High risk (score 3-6)	13 (65%)	18 (90%)	6 (30%)

**Figure 1:**
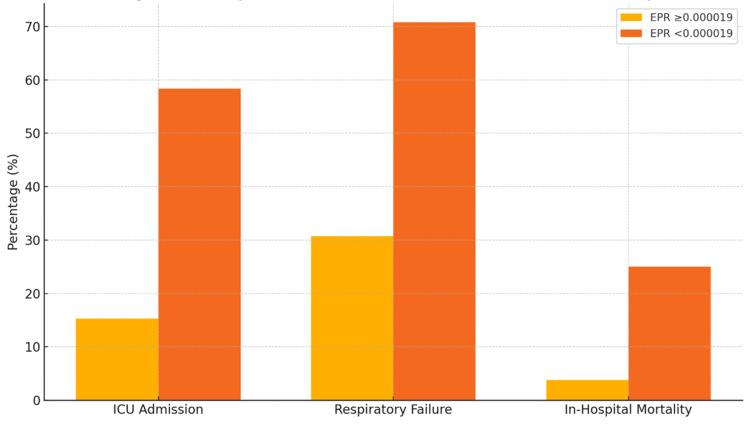
Clinical outcomes stratified by EPR threshold (0.000019) Bar chart showing frequency of major outcomes between high-EPR group (≥0.000019, n = 26) and low-EPR group (<0.000019, n = 24). Statistical significance determined using the χ² test or Fisher's exact test (p < 0.05). EPR: eosinophil-to-platelet ratio, ICU: intensive care unit

ICU admissions were also more frequent in the low-EPR group. A total of 14 out of 24 (58.3%) patients in Group B required ICU care, compared to only four (15.3%) patients in Group A. The need for ventilatory support was similarly higher in the low-EPR group. The overall distribution of clinical outcomes, including ICU admission, ventilator requirement, and survival, between the two groups is depicted, as given in Figure 1.

Across the entire cohort, patients with EPR < 0.000019 had significantly worse outcomes: higher DECAF scores, greater incidence of respiratory failure, more frequent ICU admissions, and higher mortality. No significant differences were observed in comorbidities, age, or gender distribution between the two groups, indicating that EPR served as an independent predictor of poor prognosis.

## Discussion

Our study set out to determine whether the eosinophil-to-platelet ratio (EPR) could stand on its own as a bedside prognostic tool during acute COPD, with the DECAF score. Our results demonstrate a significant association: patients with EPR below 1.9 × 10⁻⁵ had higher in-hospital mortality, increased hypercapnic respiratory failure, elevated DECAF scores, and more frequent ICU admissions. However, these findings must be interpreted in the context of multiple contributing factors. Baseline disease severity (GOLD stage and forced expiratory volume in 1 second (FEV1)), prior exacerbation frequency, medication adherence, and comorbidity burden all influence AECOPD outcomes. While our data show a strong correlation between low EPR and adverse outcomes, the observational nature of this study and the absence of multivariable adjustment prevent us from establishing EPR as an independent predictor. EPR appears to reflect a confluence of immunosuppression and inflammatory burden that correlates with, but may not independently drive, poor prognosis.

A low eosinophil count has worse outcomes in COPD and other critical illnesses. Prior work shows that suppressed eosinophil levels accompany severe infection, systemic inflammation, and poorer prognosis in both AECOPD and sepsis [11,20]. The ratio's independent tie to mortality mirrors earlier reports that connect low eosinophil counts with ICU deaths and prolonged hospital stays in COPD exacerbations [22,23].

Platelet indices have also emerged as markers of systemic inflammation and hypoxic stress. Thrombocytosis, often observed in exacerbation states, may reflect heightened inflammatory activation and altered hematopoiesis in response to cytokine release [15,16]. The inclusion of platelet count in the EPR allows for a more dynamic representation of the balance between immunosuppression (via eosinopenia) and systemic inflammatory activation (via thrombocytosis). Our findings corroborate with previous literature where platelet indices correlated with COPD severity and clinical deterioration [17,24].

The significant association between EPR and DECAF scores in this study further supports the relevance of EPR in clinical practice. Nearly 80% of patients with low EPR fell into the high-risk DECAF category, underscoring its congruence with a well-established mortality predictor. This relationship is noteworthy because, unlike the DECAF score, which requires clinical judgment (dyspnea assessment), radiographic interpretation (consolidation), and arterial blood gas analysis, EPR can be rapidly calculated from routine hematologic data, even in emergency or resource-limited settings. Several earlier studies have emphasized the prognostic value of eosinophil counts alone in COPD, with higher eosinophil levels being associated with better steroid responsiveness and lower mortality [14,23]. However, EPR adds further prognostic depth by incorporating the inflammatory burden reflected through platelet count. Notably, the ROC analysis in our study yielded a sharp discrimination threshold (0.000019), allowing objective stratification of patients based on outcome risks.

Despite the clinical relevance of our findings, several limitations must be acknowledged. First, the study's design limits the generalizability of our findings. The sample size was relatively modest (n = 50), and the study was conducted at a single tertiary care hospital in Bengaluru, India. This setting may not represent community hospitals or other geographic regions with different population characteristics and healthcare infrastructure. Furthermore, the cohort was predominantly male patients (72%), reflecting local smoking patterns but limiting applicability to female COPD populations. The study duration of four months (March-June 2025) also introduces potential seasonal bias, as COPD exacerbations show variation related to viral respiratory infections and air quality. Before EPR can be recommended for widespread clinical adoption, validation in multicenter, geographically diverse cohorts with balanced gender representation, and longer recruitment periods spanning multiple seasons is essential.

Two other significant limitations concern the scope of our analysis. An important limitation is the focus solely on in-hospital outcomes without systematic post-discharge follow-up. AECOPD prognosis extends beyond hospitalization, with 30-day readmission rates and 90-day mortality being important clinical endpoints. Future studies should incorporate prospective follow-up to assess whether low EPR values at admission predict post-discharge adverse events, readmissions, and long-term mortality. Additionally, this study lacks a multivariable analysis to adjust for potential confounding factors. Variables such as baseline COPD severity (GOLD stage and FEV1), prior exacerbation frequency, and comorbidity burden are known prognostic factors. Without multivariable logistic regression modeling, we cannot definitively establish whether EPR is an independent predictor or whether it functions as a surrogate marker for overall disease severity. Future studies should employ multivariable models to determine its independent prognostic value.

Finally, while EPR shows promise as a prognostic tool, these limitations underscore that biomarkers, including EPR, should complement rather than replace clinical assessment and established scoring systems like DECAF. Laboratory parameters provide objective, quantifiable data, but their interpretation requires integration with clinical context, including patient symptoms, physical examination findings, and comorbidities. The heterogeneous nature of COPD means that no single biomarker can capture the full spectrum of disease severity. Clinical experience remains crucial in identifying patients who may have misleading laboratory values due to concurrent conditions, medications, or other confounding factors. For instance, patients on corticosteroids or with concurrent infections may have altered eosinophil counts, and conditions affecting platelet counts can influence the EPR calculation. Therefore, EPR should be viewed as an additional tool in the clinician's armamentarium, particularly valuable for initial triage, but always requiring validation through comprehensive clinical evaluation. The combination of biomarker data with structured clinical scores and physician judgment creates a more robust framework for decision-making in AECOPD management.

In summary, this study supports the eosinophil-to-platelet ratio as a significant and independent predictor of mortality, ICU admission, and respiratory failure in patients hospitalized with acute exacerbation of COPD. Its strong correlation with DECAF score categories, coupled with ease of computation and universal accessibility, suggests that EPR could be integrated into early risk stratification protocols.

## Conclusions

The study highlights the eosinophil-to-platelet ratio (EPR) as a rapid, accessible, and cost-effective biomarker for predicting clinical severity and outcomes in patients with AECOPD. It offers significant utility in early triage and decision-making, especially in resource-limited settings where access to advanced diagnostics may be delayed. Incorporating EPR into routine evaluation can help identify high-risk patients who may benefit from intensive monitoring and timely interventions. Because EPR requires no extra blood draw, no expensive cartridge, and no specialized software, just the same laboratory slip that prints out hemoglobin and white cell counts, it can be calculated within minutes of admission. Patients with AECOPD with EPR less than 0.000019 were associated with increased risk of mortality, hypercarbia, and ICU admission.
